# The Application of Montmorillonite (MMT), Halloysite (HNT), and Carbon Nanotubes (CNT) in Toughened Polyethylene Terephthalate Glycol/Polycarbonate (PETG/PC) Blends: The Critical View on the Use of Nanosized Fillers as Phase Structure Modifiers

**DOI:** 10.3390/polym17111463

**Published:** 2025-05-25

**Authors:** Mateusz Markowski, Adam Piasecki, Jacek Andrzejewski

**Affiliations:** 1 Faculty of Materials Engineering and Technical Physics, Poznan University of Technology, Piotrowo 3 Str, 60-965 Poznan, Poland; mateusz.mm.markowski@gmail.com; 2 Institute of Materials Engineering, Faculty of Materials Engineering and Technical Physics, Poznan University of Technology, Piotrowo 3 Str, 60-965 Poznan, Poland; adam.piasecki@put.poznan.pl; 3 Institute of Materials Technology, Faculty of Mechanical Engineering, Poznan University of Technology, Piotrowo 3 Str, 61-138 Poznan, Poland

**Keywords:** polymer blends, polycarbonate, polyethylene terephthalate glycol, additive manufacturing, structure properties correlation, nanocomposites, nanoclay, carbon nanotube

## Abstract

The subject of the conducted study was primarily focused on the development of a new type of polymer blend modified with the use of nanosized fillers. The research concept involved the use of polycarbonate/polyethylene terephthalate glycol (PETG/PC) blends modified with the EBA-GMA impact modifier (ethylene–butylene–acrylonitrile copolymer) and three different types of nanofillers: montmorillonite (MMT), halloysite (HNT), and carbon nanotubes (CNT) of two types. The combination of PC, PETG, and EBA phases was used in order to achieve enhanced mechanical performance and stable processing properties. The results of the conducted study revealed that for the toughened PETG/PC/EBA blends, the impact resistance was strongly improved from the reference by 1.5 kJ/m^2^ to 15 kJ/m^2^. However, the results for the nanocomposites revealed that the MMT and HNT additions were limiting the impact strength. In contrast, the Charpy test results for CNT were again close to 15 kJ/m^2^. The results of the thermal resistance measurements again revealed more favorable properties for CNT-modified PETG/PC/EBA blends.

## 1. Introduction

Since the beginning of their application, polymeric materials have been subjected to mixing [1,2,3,4]. Considering many factors, the melt blending technique was identified as the most effective method of polymer modification. Although many polymer systems are immiscible or form an incompatible system, the appropriate selection of compounding process conditions or introducing compatibilizers usually leads to significant improvement in the structural performance [5,6,7,8]. At present, the use of polymer blends is strongly combined with different types of mass production manufacturing. For example, styrene-based materials are typically used in applications requiring low surface quality and impact strength, while polyamide blends are used in high-temperature conditions. Unfortunately, in the case of polymer blends, there are numerous obstacles to the compatibilization of the structure of such materials. In particular, the main problem is the lack of miscibility resulting from the poor interactions at the phase boundary. Usually, an attempt to improve a selected feature of a polymer ends with no significant improvement and a deterioration of the remaining properties. A popular concept to solve these limitations is to use a sufficiently high concentration of technical polymer additives, which would consequently improve the properties of the final blend. Unfortunately, numerous examples of works where PC, ABS, or PA11 additives were used indicate that the effective content of the additional component should exceed 50%, and it is often necessary to use impact modifiers [9,10,11,12,13,14]. Hence, a key aspect for further development in the field of polymer blend materials is the development of polymer systems for which a change in material properties will not require the replacement of the original component of the blend.

In the case of the discussed work, it is worth paying attention to certain specific requirements for materials processed using the filament-based 3D-printing technique. Materials of this type, apart from the obvious utility requirements for finished products, must meet certain parameters critical to the processing process itself. First of all, polymer materials dedicated to filament production should be characterized by a low melting temperature and appropriately low viscosity. While for most FDM devices, the melting process can be carried out even above 250 °C, in the case of highly filled materials, the process of plasticizing the material can be very difficult due to the lack of additional pressure and shear forces as in screw systems. The second crucial feature is associated with the low shrinkage requirements. As a result, the most popular polymer varieties intended for FDM processing are currently PLA, ABS, and PETG, with the latter polymer having been gaining particular popularity over the past few years. In previous studies, the subject of PETG-based blends preparation was rarely taken up; an example may be an attempt to produce mixtures based on the PETG/PE system [15], or PETF/PEF [16]. For materials intended for FDM printing, there were few examples of the PETG/PET blends [17,18,19]. The results of most studies indicate that, due to its amorphous nature, the structure of PETG is difficult to modify; the addition of other polymers does not support the formation of the crystalline phase, as can be observed for semicrystalline PLA. However, most of the studies suggest that PETG can form partially miscible blend systems with other polyesters. An interesting example here is the PETG/PC polymer system [20,21]. The research conducted for this type of material aimed to produce materials characterized by an undemanding processing process, and mainly low plasticization temperature and low viscosity, for which the PETG component was responsible. The addition of PC was intended to improve impact strength and thermomechanical properties. This concept, therefore, constituted the starting point for the work discussed in the article. Due to the fact that for materials in the two-component PETG/PC system, it is not possible to change the mechanical characteristics to a more ductile one; therefore, in the modified system, the additional component is an elastomeric impact modifier (IM). It is worth pointing out that the effectiveness of using this type of material depends on the content of the elastomeric phase. Usually, the addition of about 20–30% IM is found to be an effective concentration to improve impact strength and elongation. Previous studies confirmed that the toughening compound, which was an ethylene–butyl acrylate copolymer grafted with glycidyl methacrylate (EBA-GMA), can be successfully used for polyester-based blends [22,23], and also for materials intended for FDM printing [24,25,26].

In addition to the material toughening procedure, the developed blends were simultaneously subjected to modification with the use of nanofiller additives. The nanoparticles can be used as modifiers in many aspects of polymer processing, from the reinforcement purpose in composites [27,28,29] through their use as barrier additives in foils/coatings [30,31,32], ending with the use of nanoparticles to improve thermal or electrical conductivity [33,34,35]. Nanoparticles can be differentiated according to many factors, such as size, morphology, or mode of reception. However, there are two groups of nanosized materials that are investigated most often. The first group consists of nanoclays, mostly in the form of nanoplates, nanoneedles, or nanospheres. The second group is nanocarbon particles, which occur in many morphological varieties, like spherical fullerenes [36,37], nanofibers/nanotubes [38], or nanosheets (graphene) [39,40]. The nanoparticles are actually modified with different types of organic compatibilizers [41,42,43]. In general, this topic is covered in a huge number of publications. The effectiveness of this type of material has been confirmed for both mineral particles and nanotubes, but due to the multitude of available materials, it is not possible to purchase materials based on MMT, HNT, and CNT compatibilized with the same type of compounds. Hence, the concept presented in this work assumes conducting preliminary studies using unmodified additives.

The intention of this research is to combine the concept of modifying polymeric materials using a complex system of nanofillers and elastomeric impact modifiers. In selected works where such a system was implemented, favorable changes in the morphology of the elastomeric phase led to a significant increase in impact strength, which was the primary objective of this procedure [44,45,46].

To determine whether the aforementioned modification mechanism can be applied to polymer blend systems, four types of nanoadditives were tested: two nanometric clay varieties, MMT and HNT, and two types of carbon nanotubes that differ in length. The basic polymer system is a mixture of PETG/PC (50/50%) modified with the addition of 20% EBA-GMA copolymer. For all prepared blends, the nanofiller content was kept constant at 1%. Since the prepared blends are intended for FDM printing, sample preparation involved extruding the filament rod and 3D-printing the necessary specimens. The quality of the printing was tested using a complex geometry 3D model. The prepared materials underwent a comprehensive cycle of material properties characterization, including static tensile tests, Charpy impact measurements, thermomechanical analysis using the dynamic mechanical analysis (DMTA) method, and heat deflection temperature tests (HDT). The structure of the developed blends was examined using scanning electron microscopy (SEM). The selected model can serve as an example of a technical product due to the presence of holes, perpendicular walls, and inclinations. 

## 2. Experimental

### 2.1. Materials

The filament materials necessary for preparing the test samples were created through a multi-step process, which involved drying the ingredients, mixing them in a twin-screw extruder, producing the filament with a single-screw extruder, and 3D-printing the samples using the FDM method. PC and PETG pellets, along with various fillers and additives, were utilized to produce the samples:

- Unmodified polycarbonate resin Macrolon PC 2205 (BASF, Ludwigshafen, Germany, supplied by Albis Polska(Poznan, Poland)), with a melt flow rate (MFR) of 37 g/10 min (1.2 kg/300 °C)
- Poly(ethylene terephthalate)-glycol PETG resin Eastar 6763 (Eastman Chemicals, Kingsport, TN, USA), inherent viscosity (IV) = 0.70 dL/g;
- Ethylene–butyl acrylate copolymer grafted with glycidyl methacrylate EBA-g-GMA toughening agent Elvaloy PTW (Du Pont, Wilmington, DE, USA), MFR = 12 g/10 min (2.16 kg/190 °C), 28% acrylate comonomer content;
- Multi-walled carbon nanotubes CARBON4nano (Institute of Carbon Technology, Poland), with lengths of 1–2 μm (CNT-short) and 5–20 μm (CNT-long);
- Halloysite nanoclay (Sigma-Aldrich, Burlington, MA, USA)—kaolin clay in the form of nanotubes with lengths of 1–3 μm;
- CLOISITE-20 A montmorillonite (BYK, Wesel, Germany)—a nanoclay with a structure of Lamellar;
- Joncryl 4368C (BASF, Ludwigshafen, Germany)—chain extender, poly(styrene-acrylic-co-glycidyl methacrylate), supplied in the white powdered form.

Before processing, the PETG and PC pellets were dried in separate cabinet dryers for 12 h; the temperature was set to 70 °C and 100 °C, respectively, for PETG and PC. The compounded blends were dried at 70 °C. The limited drying temperature was due to the softening of the amorphous polyester phase.

### 2.2. Sample Preparation

Before the melt compounding process, all the ingredients were mixed in the dry state using the rotary mixer. The dry-blend prepared in this way was placed in the hopper of a twin-screw extruder, a model of the ZAMAK EH 16.2D type (Zamak Mercator, Skawina, Poland), and further mixed in a molten state. The maximum temperature of the mixing process was 280 °C, while the screw speed was 100 rpm and the extrusion output was about 2 kg/hour. After leaving the extruder head, the materials were cooled in a stream of cold air and granulated using a milling granulator.

The preparation stage of the filament, from which the samples for the tests were later printed, was carried out using a METALCHEM 20–32D-type single-screw extruder (IMPiB, Torun, Poland). The maximum extrusion temperature measured at the extruder die-head was 270 °C, while the screw speed was set to 18 rpm. The diameter of the extruder nozzle was 2 mm, and the target filament diameter of approximately 1.75 mm was achieved by adjusting the extraction speed accordingly, which ranged from 6 to 8 m/min depending on the viscosity of the melt. Cooling of the filament occurred in a stream of cold air. The finished material was wound onto spools using a winder. The full list of sample designations and material/filament formulations is provided in Table 1.

**Figure 1:**
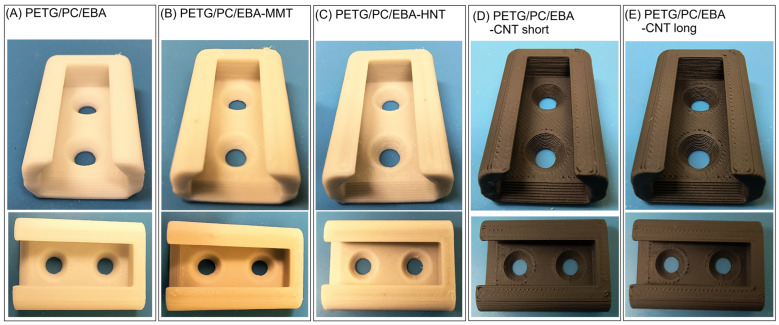
The appearance of the complex shape parts after the FDM printing procedure: (**A**) PETG/PC/EBA, (**B**) PETG/PC/EBA-MMT, (**C**) PETG/PC/EBA-HNT, (**D**) PETG/PC/EBA-CNT-short, and (**E**) PETG/PC/EBA-CNT long.

The process of printing the test specimens was conducted using a MEX/FDM-type printer, model Prusa MK3S (PrusaResearch, Czech Republic). The nozzle temperature was uniform for all composites, set at 275 °C. The table temperature was also consistent for all materials, established at 110 °C. The printing speed was 80 mm/min for the infill area and 40 mm/min for the shell/perimeter layer. The samples were printed with 100% fill, and all samples were prepared with 2 layers of shell perimeter. The STL files in the Prusa Slicer software were used to generate the G-code files (machine code). The printing quality of complex parts was evaluated throughout the printing procedure. The model used in the tests can serve as an example of a technical product due to the presence of holes, perpendicular walls, and overhangs. The macro view comparison for all prepared samples is shown in Figure 1. The appearance of the samples indicates no negative phenomena during the printing process; the bottom part of the product is flat and did not detach from the bed table during printing. The model layers did not delaminate, and the surface of the sample is even. There are also signs of stringing, which suggest problems with low viscosity typical of PETG. Therefore, it can be stated that, in the case of the obtained PETG/PC/EBA mixtures, optimal processing properties for printing were achieved, and further work on this topic is feasible.

### 2.3. Characterization Methods

Mechanical tests were performed using the following measurement methods: static tensile tests and Charpy impact tests. The tensile test was conducted on a Z010 machine (Zwick/Roell, Germany), with the cross-head speed set to 10 mm/min. The tests were executed on type 1A specimens according to the ISO 527 standard [47].

The impact strength of the samples was evaluated using the Charpy method, where rectangular samples (80 mm × 20 mm × 4 mm) were notched to a depth of 2 mm. Tests were carried out using the Zwick/Roell HIT25 (5 J pendulum), in accordance with the ISO 179 standard [48].

Dynamic thermomechanical analysis (DMTA) was performed using a MCR 301 rotational rheometer (Anton Paar, Austria) equipped with torsion clamps. Measurements were conducted within the 25 to 180 °C temperature range at a heating rate of 2 °C/min. The strain frequency was set to 1 Hz, and the measured strain value was 0.01%. Results are presented as storage modulus and tan δ plots. The thermomechanical properties were assessed using the heat deflection method (HDT). Tests were carried out with two different load sets, 0.455 MPa and 1.8 MPa, following the ISO 75 standard [49]. The measurements utilized a Vicat/HDT apparatus, model HV300C TestLab (Warsaw, Poland). Samples were immersed in an oil bath, and the heating rate for all measurements was 2 K/min.

## 3. Results

### 3.1. Structural Appearance—Optical Analysis/Scanning Electron Microscopy

**Figure 2:**
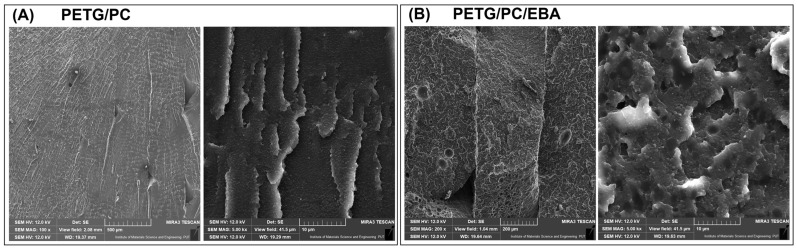
The structural appearance of the reference (**A**) PETG/PC blend and (**B**) PETG/PC/EBA.

**Figure 3:**
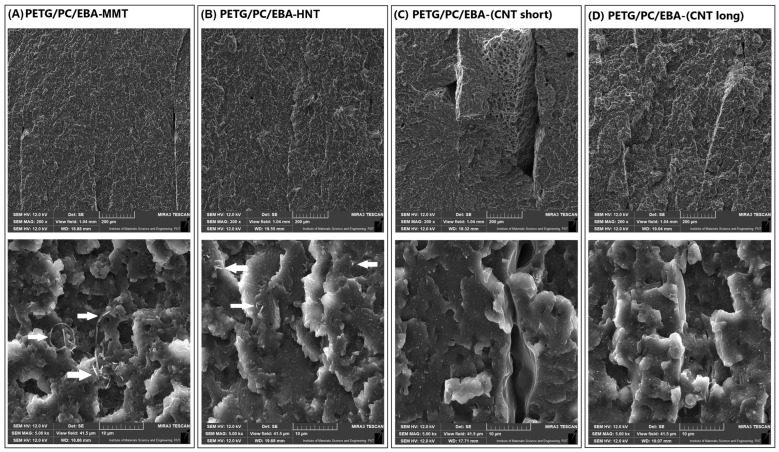
Microscopic views of the PETG/PC/EBA blends modified by nanofillers: (**A**) MMT, (**B**) HNT, (**C**) CNT-short, and (**D**) CNT-long.

The structure analysis for the prepared materials was investigated using the scanning electron microscope method. The comparison of the unmodified PETG/PC blend with the EBA elastomer-modified sample is presented in Figure 2, while the structure of materials with nanofillers is revealed in Figure 3.

It is clear that the addition of an elastomeric phase strongly influences the appearance of the microstructure, which has been confirmed by numerous research papers in which the EBA-g-GMA copolymer and related copolymers were introduced into the matrix [50,51,52]. The spherical inclusions are easy to distinguish, even given the two-phase structure of the matrix system used. The PETG/PC blend structure is presented in Figure 2. Other research has already described a highly uniform and homogeneous structure [20,21]. The smooth surface of the fracture at low magnification (×100) confirms the brittle behavior of the prepared blend, while the high magnification view (×5k) reveals that the two-phase structure is highly dispersed. The average size of the phase inclusion is significantly below 1 µm, confirming the self-compatibilization of the PETG-PC blend system. Compared to the fine dispersion of the matrix system, the EBA inclusion size ranges from 1 µm to 3 µm, which can be considered relatively large, suggesting a lack of compatibility; however, for most rubber-toughened materials, this is a typical type of structure. The mechanical test results for molded samples also confirm that the effectiveness of the elastomer used is very good.

The structure of the nanoparticle-modified samples is presented in Figure 3. The low magnification view of the fracture cross-section reveals uniform roughness across all modified materials. The absence of visible differences was also noted in the high magnification view, where the extensive fracture structure with discernible elastomer phase inclusions indicates a change in the deformation mechanism, particularly when compared to the pure PETG/PC blend. Interestingly, the presence of nanoparticles in the MMT/HNT and CNT additives is not revealed in the same manner. For the mineral type of particles, the presence of filler particles is reflected in an inhomogeneous filler distribution, with clusters of MMT plates and HNT needles concentrated together. This behavior is unfavorable as the effectiveness of the nano-type fillers is significantly reduced when particle distribution is not optimal. In this case, it is challenging to identify the substantial agglomeration of nanoparticles, since their clusters are small, and their morphology indicates complete exfoliation of the structures of individual MMT plates and HNT needles. Different trends are observed with the CNT nanoparticles, where well-distributed single nanofibers can be distinguished across the entire surface of the sample for both short and long types of CNT particles. It appears that the morphology of the carbon nanofiber-modified materials is more favorable than that of the other nanocomposites; however, aside from the visible differences in particle distribution, the observations do not reveal significant differences between the toughened materials.

### 3.2. Mechanical Performance—Static Tensile Test/Charpy Impact Measurements

**Figure 4:**
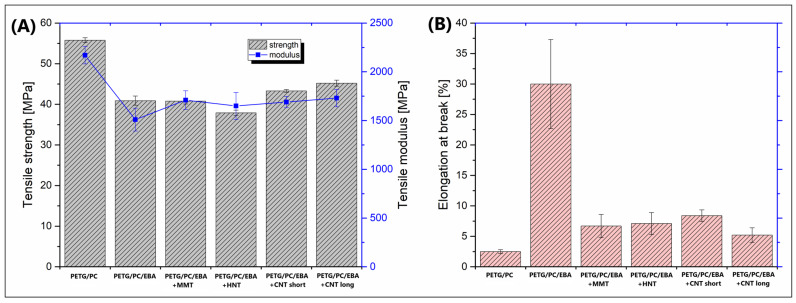
The results of the static tensile tests for extruder filament rod samples: (**A**) tensile strength/modulus; (**B**) elongation at break.

**Figure 5:**
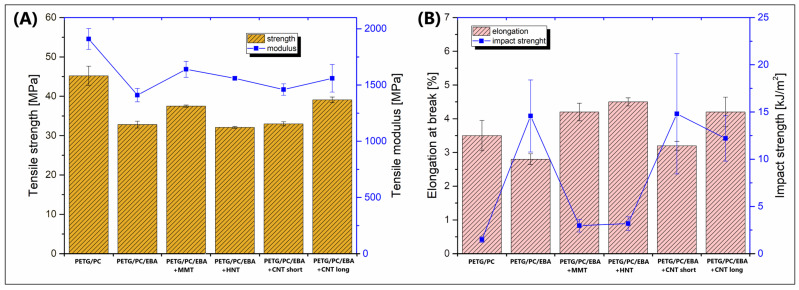
The results of the static tensile and Charpy impact measurements: (**A**) tensile strength/modulus; (**B**) elongation at break/impact strength.

The study focused on evaluating the material properties of MEX (FDM) printed samples; however, for reference purposes, a series of tensile tests were also conducted using filament-based specimens. The results of this preliminary study are presented in Figure 4. The mechanical properties of the 3D-printed samples are shown in the plots in Figure 5.

Since the filament-based specimens reflected the properties of solid samples, we can assume that most properties of this type of material will be similar to those of injection/compression molded parts. Clearly, we can also conclude that the expected properties can be significantly better than those of printed samples. The main factor visibly influencing the strength and modulus of the recorded results is the presence of an elastomeric compound. The initial tensile modulus value for the PETG/PC blend was around 2200 MPa, while for PETG/PC/EBA, it was only 1500 MPa. A similar decrease was observed in the tensile strength values, with recorded differences of 56 MPa and 41 MPa, respectively, for the unmodified blend and the EBA-modified material. Interestingly, the addition of nanofillers (MMT, HNT, CNT) does not lead to significant changes in tensile properties, as most recorded results are very close to those of the PETG/PC material. The results from the elongation-at-break measurements are even more intriguing. As predicted, the fastest fracture was observed in the unmodified PETG/PC blend (≈2.5%). Adding a 20% EBA modifier resulted in significant improvement, with the maximum strain reaching around 30%. Despite using the same amount of the elastomeric phase, the elongation of the nanofiller-modified materials was noticeably reduced. Therefore, we can conclude that adding even a small amount of nanoparticles greatly influences the reinforcing efficiency, or that the phase structure is significantly altered.

The presented results indicate a significant difference in tensile modulus between the unmodified PETG/PC blend and samples with the EBA copolymer addition. The initial stiffness of the PETG/PC material was close to 1900 MPa, while the addition of the elastomer decreased this value to 1400–1700 MPa, with the lowest modulus recorded for the PETG/PC/EBA sample (1410 MPa). Interestingly, for the nanofiller-modified samples, there was no apparent trend indicating the reinforcing efficiency of fibrous particles. The highest tensile modulus was recorded for the MMT-modified blend (1640 MPa). Similar changes are observed when analyzing the tensile strength results; the values for the pure PETG/PC blend were the highest (≈45 MPa), while those for the EBA elastomer and EBA-/nanofiller-modified samples were noticeably lower. It can be concluded that the addition of the EBA impact modifier was the primary factor influencing the strength/modulus correlations for the prepared materials. More noticeable changes that might suggest differences in nanofiller–matrix interactions are observed when analyzing the impact strength and elongation-at-break results. The recorded values of maximum strain rate were surprisingly low for the 3D-printed samples, as none of the samples reached 5% elongation. Such results indicate the brittle behavior of the developed materials.

### 3.3. Thermomechanical Performance—Dynamic Mechanical Thermal Analysis/Heat Deflection Temperature (DMTA/HDT)

**Figure 6:**
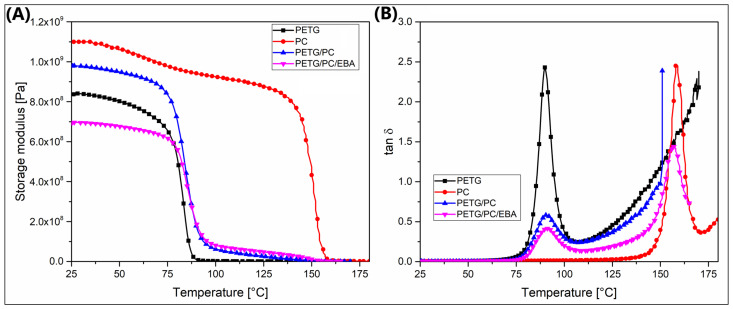
The DMTA analysis results for the reference materials: (**A**) storage modulus, (**B**) tan δ plots. The collected plots present the results for pure PETG, pure PC, the PETG/PC blend, and the PETG/PC/EBA blend.

**Figure 7:**
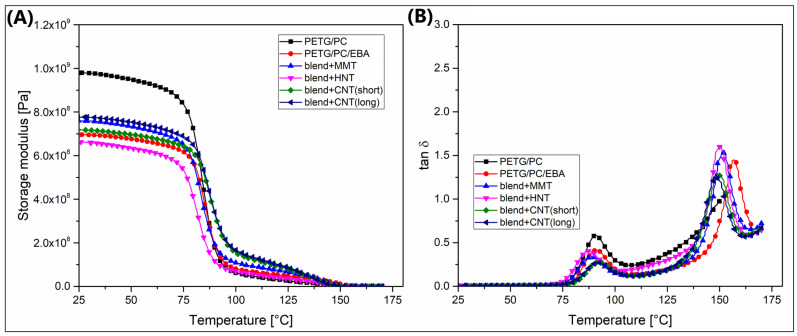
The comparison of the DMTA results for the MMT-, HNT-, and CNT-modified blends: (**A**) storage modulus, (**B**) tan δ.

The DMTA analysis was conducted according to a standard procedure, where the results of the measurements are presented in the form of plots of the storage modulus and the tangent of the loss angle δ (tan δ). For reference materials, the plots are presented in Figure 6, while the results for the nanofiller-modified samples are collected in Figure 7.

Measurement results for the reference materials PETG, PC, and the mixtures of PETG/PC and PETG/PC/EBA presented below confirm the distinctly different thermomechanical characteristics of the base polymers. The true modulus value for PETG drops dramatically around 70 °C, indicating a low softening temperature. In contrast, the stiffness of PC remains stable even at 130 °C, with a significant decrease in the true modulus observed only around 150 °C. Interestingly, despite the ratio of the two polymers in the base blend being 50/50 %, the thermograms of the measurements suggest that the changes in stiffness of the PETG/PC blend are more similar to the characteristics of the PETG copolymer. Although the addition of the impact modifier EBA-GMA lowers the value of the true modulus, it does not change the overall characteristics displayed in the graph. This observation is also supported by the tan δ plots, where for both varieties of polymer blends, the positions of the peaks responsible for the relaxation of both the PETG phase and the PC phase did not shift, confirming the lack of miscibility of the obtained system.

The results for the mixtures modified with nanoparticles are presented in a similar manner. For comparative purposes, in addition to materials modified with the EBA-GMA elastomer, the graphs also include outcomes for the PETG/PC mixture. As expected, the stiffness of the reference sample is significantly higher than in the other systems. However, the differences in stiffness among the various types of nanoadditives are quite negligible and appear random. Some more logical relationships emerge in the tan δ plots. The first peak near 90 °C, corresponding to the relaxation of the PETG phase, shows that the greatest reduction in the area under the peak occurs for samples containing both types of CNTs, while the changes for the MMT and HNT particles are quite minimal. Similar correlations are observed for the second peak related to the PC phase relaxation. Again, the addition of CNTs results in a decrease in the peak area, clearly indicating a higher macromolecular structure entanglement factor for this class of additives. Interestingly, a visible difference is noted between the PETG/PC/EBA and PETG/PC/EBA + nanofillers samples, where the position of the PC phase peak shifts to a lower temperature. A possible explanation for this visible difference is phase separation due to the presence of a functionalized elastomeric phase; however, the observed changes are more pronounced for the hybrid (elastomer/nanofiller) systems, suggesting that the nanoparticles tend to locate in the interphase or penetrate between phase boundaries, leading to alterations in the position of the relaxation temperatures for the individual phases.

**Figure 8:**
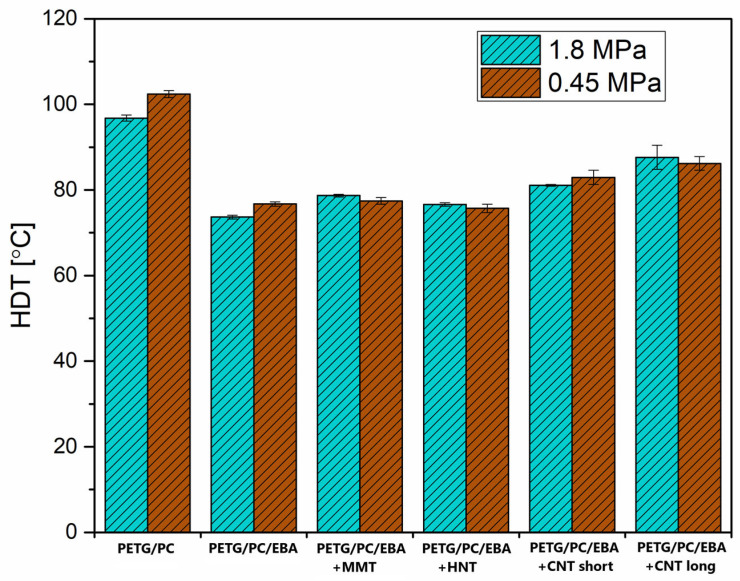
The results of the heat deflection temperature (HDT) measurements. Tests were conducted using 1.8 MPa and 0.455 MPa loads.

Some correlation in thermomechanical properties is easily confirmed when comparing the DMTA results with industrial measurements (see Figure 8), particularly regarding heat deflection temperature measurements (HDT). The measurements were performed under two different loading conditions: 0.45 MPa and 1.8 MPa. This approach allows for assessing the influence of the applied force on the resulting deflection temperature. For the reference PETG/PC sample, the recorded HDT temperature was the highest, reaching around 100 °C. As anticipated, there was a noticeable difference between the 0.45 MPa and 1.8 MPa loads, which were 102 °C and 96 °C, respectively; however, this difference cannot be considered significant. When compared to the reference blend, the HDT test results of PETG/PC/EBA deteriorate significantly, ranging from 74 °C to 78 °C. The addition of MMT and HNT particles does not result in any noticeable change in thermomechanical resistance; interestingly, for both materials, the HDT values were higher with the 1.8 MPa load. Although this behavior is unexpected, given that the recorded difference does not exceed 2 °C, it can be concluded that it falls within the margin of measurement error. The results for the CNT-modified samples showed a slight increase compared to the PETG/PC/EBA blend. The best result for the short-type CNT-modified materials was around 83 °C, while for the long CNT particles, it was close to 88 °C. The HDT measurement results confirm that the most effective mechanism for structure reinforcement is observed with fibrous types of nanofillers. Additionally, the length of the used CNT also influences the structural interactions, leading to enhanced thermal resistance.

## 4. Discussion

The expectations regarding the applied nanomaterials turned out to be somewhat disappointing, which, to some extent, can be considered a very positive result, as it verifies certain top-down assumptions regarding the effectiveness of using nanomaterials in practical applications. First of all, the results indicate the low efficiency of nanoclay-based materials. The mechanical test results are surprisingly low for all tested samples, even when compared to those of the reference PETG/PC samples. The main possible explanation for that behavior is the occurrence of visible problems with the agglomeration of the MMT and HNT particles. In most of the SEM images taken at high magnification, agglomerates of these nanoparticles are visible. Surprisingly, in the case of the CNT particles, both the long and short nanotubes, SEM imaging indicates very good dispersion, no agglomerates, and much better mechanical performance results. This behavior is quite surprising, given the potentially greater tendency of fibrous materials, such as nanotubes, to form agglomerated structures. The CNT fibers are usually supplied in the form of entangled bundles, where the proper homogenization with thermoplastic polymer is very difficult [53]. The studies conducted so far indicate that in most cases, the problem of CNT particle dispersion is a major technological problem dependent on many factors. However, due to the high efficiency of the structure reinforcement by even a small amount of nanotubes, the properties of materials modified in this way change significantly, which is manifested primarily in mechanical measurements [54]. Even if the CNT dispersion is incomplete, the effectiveness of the nanotubes distributed throughout the matrix is usually sufficient to obtain a visible change in the material characteristics. However, when analyzing the situation regarding the addition of nanoclays again, the key aspect that should be considered here is the lack of surface modification for the materials used. Previous studies, including those covering materials manufactured using the FDM technique [42,55,56], indicate that the use of systems surfaces modified with organic compounds brings much better results than the use of unmodified mineral nanoadditives [27,57,58].

The next phenomenon analyzed in the work is the behavior of the structure of polymer mixtures in the presence of nanoadditives [59,60]. The initial assumptions regarding the preparation of the tested materials assumed the possibility of localizing nanoadditives in one of the phases of the processed material. For example, studies of this type conducted on LDPE/PLA mixtures indicated the localization of CNTs in the LDPE phase, which led to an increase in electrical conductivity and for the tested materials [61]. In another case, the addition of nanosilica in the polyamide6/polystyrene mixture system led to changes in the morphology of the dispersed phase, which consequently allowed for maintaining the lamellar nature of the tested system [62]. In the case of the discussed studies, the expected phenomenon was the modification of the PETG/PC matrix morphology phase towards obtaining a co-continuous phase. This phenomenon was already described for PLA/PA11 and PMMA/PS blends modified by MMT filler [63,64]. As it turns out, for the materials tested, the process of changing the morphology of the phase system of the mixture turned out to be very difficult, which could have been influenced by several factors. First of all, the systems analyzed so far were characterized by very low miscibility, an example of which is the PLA/PA11 system, where a significant size of the dispersed phase inclusion suggests a lack of effective physical or chemical interactions at the phase boundary. On the other hand, in the case of the PETG/PC-type material obtained during the discussed studies, despite the two-phase structure confirmed in the DMTA studies, its high level of fragmentation suggests the occurrence of certain interactions at the phase boundary, which lead to obtaining a very favorable morphology, where the size of the dispersed phase inclusion is significantly below 1 µm. Interestingly, also for the introduced elastomer phase (EBA-g-GMA), nanoadditives did not affect the changes in morphology visible in the structural analysis, which unfortunately confirms that when using such a small amount of nanofillers, changes in the morphology of the dispersed phase of the mixture are difficult to obtain. Interestingly, in the case of CNT-based materials, the beneficial changes in the impact strength values are probably the result of the pull-out mechanism, which, due to the significant degree of nanoparticle dispersion, turns out to be very effective even for a 1% CNT content, regardless of the nanotube length. It is clear that for the further studies, it will be interesting to combine the already developed methodology with more complex analyses of nanofiller dispersion [65], which will make it possible to describe the observed phenomenon in more detail.

## 5. Conclusions

The studies that were undertaken confirmed the potential application of the prepared PETG/PC blends.; However, considering the obtained results, the effectiveness of using nanofillers in the dosed amount (1 pph) seems to be of little prospect. The analysis of mechanical properties suggests a varied effect of fillers on the properties of the produced composites. The presence of the EBA-GMA copolymer had a significant effect on the mechanical properties. The samples containing its additive were characterized by only slightly lower stiffness and tensile strength. However, the positive effect of this additive was visible in the Charpy impact test. Interestingly, in this test, the highest results were obtained for the sample containing the addition of carbon nanotubes with a length of 1–2 μm; however, taking into account all the values, this was only a small improvement. The samples containing the HNT and MMT nanoclay additives seem to limit the potential provided by the elastomer additive. In their case, the results obtained in the impact test were only slightly higher than the reference sample without the EBA-GMA additive. DMTA analysis indicated the higher stiffness of the PETG/PC blends compared to pure PETG; however, for target materials with an elastomer additive (PETG/PC/EBA), the initial stiffness turns out to be the lowest. The addition of nanofillers does not significantly affect the increase in thermomechanical stability, which is visible both in the results of the DMTA analysis and HDT tests. Again, the highest improvement in thermomechanical properties was noted for the samples containing carbon nanotubes. From the point of view of further work, the most promising material is the samples with the addition of CNT. Interestingly, microscopic observations showed the very good dispersion of this additive in the applied matrix. The planned work will include the preparation and analysis of properties for materials with a minimum 5% CNT content, or in a hybrid system with the addition of carbon fibers.

## Figures and Tables

**Table 1:** The list of sample marking and material formulation.

	PETG [wt%]	PC [wt%]	EBA-g-GMA [wt%]	Chain Extender [pph *]	Nanofiller [pph]
PETG/PC	50	50	-	0.5	-
PETG/PC/EBA	40	40	20	0.5	-
PETG/PC/EBA+MMT	40	40	20	0.5	1
PETG/PC/EBA+HNT	40	40	20	0.5	1
PETG/PC/EBA+(CNT short)	40	40	20	0.5	1
PETG/PC/EBA+(CNT long)	40	40	20	0.5	1

* pph—parts per hundred.

## Data Availability

The original contributions presented in this study are included in the article. Further inquiries can be directed to the corresponding author.

## References

[1] Pötschke P., Paul D.R. (2003). Formation of Co-Continuous Structures in Melt-Mixed Immiscible Polymer Blends. J. Macromol. Sci. Polym. Rev..

[2] Nassar T.R., Paul D.R., Barlow J.W. (1979). Polyester–Polycarbonate Blends. II. Poly(Ethylene Terephthalate). J. Appl. Polym. Sci..

[3] Santos J.M.R.C.A., Guthrie J.T. (2006). Polymer Blends: The PC-PBT Case. J. Mater. Chem..

[4] Fortelny I., Ujcic A., Fambri L., Slouf M. (2019). Phase Structure, Compatibility, and Toughness of PLA/PCL Blends: A Review. Front. Mater..

[5] Yuryev Y., Mohanty A.K., Misra M. (2017). Novel Biocomposites from Biobased PC/PLA Blend Matrix System for Durable Applications. Compos. B Eng..

[6] Yuryev Y., Mohanty A.K., Misra M. (2016). Novel Super-Toughened Bio-Based Blend from Polycarbonate and Poly(Lactic Acid) for Durable Applications. RSC Adv..

[7] Lombardo B.S., Keskkula H., Paul D.R. (1994). Influence of ABS Type on Morphology and Mechanical Properties of PC/ABS Blends. J. Appl. Polym. Sci..

[8] Seelig T., Giessen E. (2007). Effects of Microstructure on Crack Tip Fields and Fracture Toughness in PC/ABS Polymer Blends. Int. J. Fract..

[9] Vadori R., Misra M., Mohanty A.K. (2017). Statistical Optimization of Compatibilized Blends of Poly(Lactic Acid) and Acrylonitrile Butadiene Styrene. J. Appl. Polym. Sci..

[10] Vadori R., Misra M., Mohanty A.K. (2016). Sustainable Biobased Blends from the Reactive Extrusion of Polylactide and Acrylonitrile Butadiene Styrene. J. Appl. Polym. Sci..

[11] Codou A., Anstey A., Misra M., Mohanty A.K. (2018). Novel Compatibilized Nylon-Based Ternary Blends with Polypropylene and Poly(Lactic Acid): Morphology Evolution and Rheological Behaviour. RSC Adv..

[12] Anstey A., Codou A., Misra M., Mohanty A.K. (2018). Novel Compatibilized Nylon-Based Ternary Blends with Polypropylene and Poly(Lactic Acid): Fractionated Crystallization Phenomena and Mechanical Performance. ACS Omega.

[13] Zhou Y., Luo L., Liu W., Zeng G., Chen Y. (2015). Preparation and Characteristic of PC/PLA/TPU Blends by Reactive Extrusion. Adv. Mater. Sci. Eng..

[14] Lin L., Deng C., Lin G., Wang Y. (2014). Mechanical Properties, Heat Resistance and Flame Retardancy of Glass Fiber-Reinforced PLA-PC Alloys Based on Aluminum Hypophosphite. Polym. Plast. Technol. Eng..

[15] Techawinyutham L., Tengsuthiwat J., Srisuk R., Techawinyutham W., Mavinkere Rangappa S., Siengchin S. (2021). Recycled LDPE/PETG Blends and HDPE/PETG Blends: Mechanical, Thermal, and Rheological Properties. J. Mater. Res. Technol..

[16] Paszkiewicz S., Irska I., Piesowicz E. (2020). Environmentally Friendly Polymer Blends Based on Post-Consumer Glycol-Modified Poly(Ethylene Terephthalate) (PET-G) Foils and Poly(Ethylene 2,5-Furanoate) (PEF): Preparation and Characterization. Materials.

[17] Bouguermouh K., Habibi M., Laperrière L., Li Z., Abdin Y. (2024). 4D-Printed PLA-PETG Polymer Blends: Comprehensive Analysis of Thermal, Mechanical, and Shape Memory Performances. J. Mater. Sci..

[18] Garwacki M., Cudnik I., Dziadowiec D., Szymczak P., Andrzejewski J. (2024). The Development of Sustainable Polyethylene Terephthalate Glycol-Based (PETG) Blends for Additive Manufacturing Processing—The Use of Multilayered Foil Waste as the Blend Component. Materials.

[19] Andrzejewski J., Chmielewski P., Osiński F., Markowski M., Marciniak-Podsadna L., Piasecki A. (2024). Use of Recycled Poly(Ethylene Terephthalate)-Based (RPET) Blends in Additive Manufacturing Techniques to Prepare Sustainable, Tough, and Heat-Resistant Parts. ACS Sustain. Chem. Eng..

[20] Andrzejewski J., Marciniak-Podsadna L. (2020). Development of Thermal Resistant FDM Printed Blends. The Preparation of GPET/PC Blends and Evaluation of Material Performance. Materials.

[21] Andrzejewski J. (2023). The Use of Recycled Polymers for the Preparation of Self-Reinforced Composites by the Overmolding Technique: Materials Performance Evaluation. Sustainability.

[22] Chang B.P., Mohanty A.K., Misra M. (2018). Tuning the Compatibility to Achieve Toughened Biobased Poly(Lactic Acid)/Poly(Butylene Terephthalate) Blends. RSC Adv..

[23] You X., Snowdon M.R., Misra M., Mohanty A.K. (2018). Biobased Poly(Ethylene Terephthalate)/Poly(Lactic Acid) Blends Tailored with Epoxide Compatibilizers. ACS Omega.

[24] Diederichs E.V., Picard M.C., Chang B.P., Misra M., Mielewski D.F., Mohanty A.K. (2019). Strategy to Improve Printability of Renewable Resource-Based Engineering Plastic Tailored for Fdm Applications. ACS Omega.

[25] Toth L., Slezák E., Bocz K., Ronkay F. (2024). Progress in 3D Printing of Recycled PET. Mater. Today Sustain..

[26] Diederichs E., Picard M., Chang B.P., Misra M., Mohanty A. (2021). Extrusion Based 3d Printing of Sustainable Biocomposites from Biocarbon and Poly(Trimethylene Terephthalate). Molecules.

[27] Zubkiewicz A., Szymczyk A., Franciszczak P., Kochmanska A., Janowska I., Paszkiewicz S. (2020). Comparing Multi-Walled Carbon Nanotubes and Halloysite Nanotubes as Reinforcements in EVA Nanocomposites. Materials.

[28] Kumar A., Sharma K., Dixit A.R. (2021). A Review on the Mechanical Properties of Polymer Composites Reinforced by Carbon Nanotubes and Graphene. Carbon. Lett..

[29] Paszkiewicz S. (2015). Adding Carbon Nanoparticles with Different Geometries to Poly(Ethylene Terephthalate). Plast. Res. Online.

[30] Nabels-Sneiders M., Barkane A., Platnieks O., Orlova L., Gaidukovs S. (2023). Biodegradable Poly(Butylene Succinate) Laminate with Nanocellulose Interphase Layer for High-Barrier Packaging Film Application. Foods.

[31] Raja Beryl J., Xavier J.R. (2021). Influence of Silane Functionalized Nanoclay on the Barrier, Mechanical and Hydrophobic Properties by Clay Nanocomposite Films in an Aggressive Chloride Medium. Colloids Surf. A Physicochem. Eng. Asp..

[32] Perera K.Y., Hopkins M., Jaiswal A.K., Jaiswal S. (2024). Nanoclays-Containing Bio-Based Packaging Materials: Properties, Applications, Safety, and Regulatory Issues. J. Nanostructure Chem..

[33] Paszkiewicz S., Taraghi I., Szymczyk A., Huczko A., Kurcz M., Przybyszewski B., Stanik R., Linares A., Ezquerra T.A., Rosłaniec Z. (2017). Electrically and Thermally Conductive Thin Elastic Polymer Foils Containing SiC Nanofibers. Compos. Sci. Technol..

[34] Bilisik K., Akter M. (2022). Polymer Nanocomposites Based on Graphite Nanoplatelets (GNPs): A Review on Thermal-Electrical Conductivity, Mechanical and Barrier Properties. J. Mater. Sci..

[35] Taraghi I., Paszkiewicz S., Fereidoon A., Szymczyk A., Stanik R., Gude M., Linares A., Ezquerra T.A., Piesowicz E., Wilpiszewska K. (2021). Thermally and Electrically Conducting Polycarbonate/Elastomer Blends Combined with Multiwalled Carbon Nanotubes. J. Thermoplast. Compos. Mater..

[36] Kitjanon J., Khuntawee W., Phongphanphanee S., Sutthibutpong T., Chattham N., Karttunen M., Wong-Ekkabut J. (2021). Nanocomposite of Fullerenes and Natural Rubbers: Martini Force Field Molecular Dynamics Simulations. Polymers.

[37] Olkhov Y.A., Jurkowski B. (2007). Effect of Fullerenes on the Structure and Properties of Linear and Crosslinked Polyesterurethane Ureas. J. Appl. Polym. Sci..

[38] Skórczewska K., Lewandowski K., Wilczewski S. (2022). Novel Composites of Poly(Vinyl Chloride) with Carbon Fibre/Carbon Nanotube Hybrid Filler. Materials.

[39] Wilczewski S., Skórczewska K., Tomaszewska J., Lewandowski K., Szulc J., Runka T. (2020). Manufacturing Homogenous PVC/Graphene Nanocomposites Using a Novel Dispersion Agent. Polym. Test..

[40] Li Y., Feng Z., Huang L., Essa K., Bilotti E., Zhang H., Peijs T., Hao L. (2019). Additive Manufacturing High Performance Graphene-Based Composites: A Review. Compos. Part A Appl. Sci. Manuf..

[41] Bouakaz B.S., Habi A., Grohens Y., Pillin I. (2017). Organomontmorillonite/Graphene-PLA/PCL Nanofilled Blends: New Strategy to Enhance the Functional Properties of PLA/PCL Blend. Appl. Clay Sci..

[42] Coiai S., Cicogna F., de Santi A., Pérez Amaro L., Spiniello R., Signori F., Fiori S., Oberhauser W., Passaglia E. (2017). MMT and LDH Organo-Modification with Surfactants Tailored for PLA Nanocomposites. Express Polym. Lett..

[43] Shibata M., Someya Y., Orihara M., Miyoshi M. (2006). Thermal and Mechanical Properties of Plasticized Poly(L-Lactide) Nanocomposites with Organo-Modified Montmorillonites. J. Appl. Polym. Sci..

[44] Banerjee R., Li Y., Ray S.S. (2025). Nanoparticle-Induced Morphology Evolution and Property Expression in Immiscible Polymer Blend Composites−A Review of Fundamental Understanding on Nanoparticle Migration and Interface Crossing. Polymer.

[45] Zhang K., Yu H.O., Shi Y.D., Chen Y.F., Zeng J.B., Guo J., Wang B., Guo Z., Wang M. (2017). Morphological Regulation Improved Electrical Conductivity and Electromagnetic Interference Shielding in Poly(l-Lactide)/Poly(ε-Caprolactone)/Carbon Nanotube Nanocomposites via Constructing Stereocomplex Crystallites. J. Mater. Chem. C Mater..

[46] Zhang J., Wu G., Huang S., Sang M., Wang H., Ye L., Ray S.S., Li Y. (2024). Wetting Kinetics-Controlled Interplay between Nanoparticles and Polymer Domains in Multiphase Polymer Blends. ACS Appl. Polym. Mater..

[47] (2012). ISO-Committee Plastics—Determination of Tensile Properties.

[48] (2010). ISO-Committee Plastics—Determination of Charpy Impact Properties.

[49] (2013). ISO-Committee Plastics—Determination of Temperature of Deflection under Load.

[50] Wu H., Hou A., Qu J.P. (2019). Phase Morphology and Performance of Supertough PLA/EMA-GMA/ZrP Nanocomposites Prepared through Reactive Melt-Blending. ACS Omega.

[51] Zolali A.M., Favis B.D. (2018). Toughening of Cocontinuous Polylactide/Polyethylene Blends via an Interfacially Percolated Intermediate Phase. Macromolecules.

[52] Chen Q., Shan P., Tong C., Yan D., Zhang Y., Liu H., Hao C. (2019). Influence of Reactive Blending Temperature on Impact Toughness and Phase Morphologies of PLA Ternary Blend System Containing Magnesium Ionomer. J. Appl. Polym. Sci..

[53] Ma P.C., Siddiqui N.A., Marom G., Kim J.K. (2010). Dispersion and Functionalization of Carbon Nanotubes for Polymer-Based Nanocomposites: A Review. Compos. Part A Appl. Sci. Manuf..

[54] Kasaliwal G.R., Pegel S., Göldel A., Pötschke Petra P., Heinrich G. (2010). Analysis of Agglomerate Dispersion Mechanisms of Multiwalled Carbon Nanotubes during Melt Mixing in Polycarbonate. Polymer.

[55] Andrzejewski J., Markowski M., Barczewski M. (2022). The Use of Nanoscale Montmorillonite (MMT) as Reinforcement for Polylactide Acid (PLA) Prepared by Fused Deposition Modeling (FDM)—Comparative Study with Biocarbon and Talc Fillers. Materials.

[56] He H., Liu B., Xue B., Zhang H. (2022). Study on Structure and Properties of Biodegradable PLA/PBAT/Organic-Modified MMT Nanocomposites. J. Thermoplast. Compos. Mater..

[57] Murariu M., Dechief A.L., Paint Y., Peeterbroeck S., Bonnaud L., Dubois P. (2012). Polylactide (PLA)-Halloysite Nanocomposites: Production, Morphology and Key-Properties. J. Polym. Environ..

[58] Rashmi B.J., Prashantha K., Lacrampe M.F., Krawczak P. (2015). Toughening of Poly(Lactic Acid) without Sacrificing Stiffness and Strength by Melt-Blending with Polyamide 11 and Selective Localization of Halloysite Nanotubes. Express Polym. Lett..

[59] Salzano De Luna M., Filippone G. (2016). Effects of Nanoparticles on the Morphology of Immiscible Polymer Blends—Challenges and Opportunities. Eur. Polym. J..

[60] Cai X., Li B., Pan Y., Wu G. (2012). Morphology Evolution of Immiscible Polymer Blends as Directed by Nanoparticle Self-Agglomeration. Polymer.

[61] Kajornprai T., Jarapanyacheep R., Saikaeo J., Pojprapai S., Jarukumjorn K., Trongsatitkul T. (2024). Double Percolation of Poly(Lactic Acid)/Low-Density Polyethylene/Carbon Nanotube (PLA/LDPE/CNT) Composites for Force-Sensor Application: Impact of Preferential Localization and Mixing Sequence. Polymers.

[62] Kong M., Huang Y., Lv Y., Wang S., Yang Q., Li G. (2014). Flow-Induced Morphological Instability in Nanosilica-Filled Polyamide 6/Polystyrene Blends. Polymer.

[63] de Luna M.S., Causa A., Filippone G. (2017). Interfacially-Located Nanoparticles Anticipate the Onset of Co-Continuity in Immiscible Polymer Blends. Polymers.

[64] Nuzzo A., Bilotti E., Peijs T., Acierno D., Filippone G. (2014). Nanoparticle-Induced Co-Continuity in Immiscible Polymer Blends—A Comparative Study on Bio-Based PLA-PA11 Blends Filled with Organoclay, Sepiolite, and Carbon Nanotubes. Polymer.

[65] Frielinghaus H., Koch K., Antonio V.P., Noda Y., Koizumi S. (2019). The Locally Columnar Model for Clay/Polymer Systems: Connections to Scattering Experiments. J. Colloid. Interface Sci..

